# Statistical Risk Characteristics and Risk Scoring of Hospital-Acquired Malnutrition for Pediatric Patients

**DOI:** 10.1155/2020/4305487

**Published:** 2020-06-09

**Authors:** Khreshna Syuhada, Dessie Wanda, Risti Nur'aini, Chairun Ardiantari, Ayu Susilo

**Affiliations:** ^1^Statistics Research Division, Institut Teknologi Bandung, Bandung, Indonesia; ^2^Faculty of Nursing, Universitas Indonesia, Depok, Indonesia

## Abstract

**Background:**

Malnutrition is a global health problem and challenge for every country. It may occur in any form and affect all levels of age including children. We pay particular attention to the so-called hospital-acquired malnutrition (HaM) for pediatric patients. Our aim was to explore statistical risk factors or characteristics as well as to forecast risk scoring for such malnutrition.

**Methods:**

This study employed a cross-sectional design involving children from 1 month to 18 years of age who were hospitalized for at least 72 hours. We used secondary data from 308 medical records of pediatric patients who were admitted to the hospital in 2017. We excluded the data if the patient had tumors or organomegaly, fluid retention, and dehydration. HaM was determined based on a weight loss each day during hospitalization until the day of discharge. Statistical data analysis is carried out for both descriptive and inferential statistics. Our predictive model is yielded by linear regression, and risk scoring is obtained through logistic regression.

**Results:**

The findings showed several risk factors or characteristics for HaM prevalence: sex, age, medical diagnosis, diet, nutrition route, and NEWS score. The early warning system to pediatric patients is conducted by calculating malnutrition-at-risk in which a value beyond 100.5 is considered as having high potential risk for HaM.

**Conclusion:**

Nurses are expected to monitor pediatric patients' condition, including measuring the anthropometry regularly, in order to identify the initial signs of HaM.

## 1. Background

Malnutrition is a broad term to describe any imbalance in nutrition, either overnutrition or undernutrition. The case of malnutrition may occur to people in the residential, see, for example, Pal [1]; Yang et al. [2]; Headey et al. [3]; and Ekbrand and Halleröd [4], and/or to patients at the hospital, e.g., de Aquino and Philippi [5]; Barker et al. [6]; Joosten and Hulst [7]; Curtis et al. [8]; Gouveia and Silva [9]; Beser et al. [10]; Maia et al. [11]; and Sanz et al. [12]. It is an interesting topic and statistically challenging from health practitioners', including nurses, and statisticians' viewpoints. In this study, we pay particular attention to the so-called hospital-acquired malnutrition (HaM) for pediatric patients in a top referral hospital in Indonesia.

Malnutrition, including malnutrition in hospital settings, remains a global issue. A study conducted by Pacheco-Acosta et al. [13] showed that 50% of patients in a hospital suffered from malnutrition. Malnutrition may occur when patients are first admitted and may worsen during their stay. According to one study conducted in Canada, 39.6% of patients from 1 month to 19 years of age who were admitted to a hospital were malnourished [14]. Such conditions could cause a deterioration of nutritional status during hospitalization.

Moeeni et al. [15, 16] have conducted studies in Iran and New Zealand and found that pediatric patients with good nutritional status tend to have malnutrition. In addition, pediatric patients with low-moderate malnutrition will have high risk of having severe malnutrition during hospitalization. This is in contrast to those with high level of malnutrition since they are monitored, observed, and evaluated kindly [15–17].

In cases of HaM, the patients show nutritional status deterioration during their hospital stay, described by weight loss [18], see also Lang et al. [19] for statistical analysis of weight change. Campanozzi et al. [20] defined HaM as the decrease of nutritional status after 72 hours of a hospital stay. Pacheco-Acosta et al. [13] determined whether a patient experienced HaM in their study when the patient's weight loss was >2% and BMI decline >0.25 SD, whilst Joosten and Hulst [7] and Villares et al. [21] claimed nutritional status decrease after 48 hours by using a BMI of 0.25 standard deviation in comparison to BMI at admission.

Based on several definitions previously mentioned, the definition of HaM in this research was determined based on the researchers' judgment since there is still no ideal method to define HaM, especially in children. Note that in any HaM settings or scenarios, quantitative health issues or problems related may be simple or complicated, see also Lang et al. [19] for statistical analysis of weight change. Consequently, statistical data analysis plays an important role particularly in determining and predicting risk characteristics or factors.

In this paper, we explore risk characteristics of the HaM case. We specifically identify factors that have significant impact on the malnutrition case. Note that these factors may be categorized as either malnutrition severity or malnutrition frequency, whilst the former emphasizes on patients' weight itself and the latter answers the question of (i) how many cases of malnutrition for certain factors and (ii) how many patients are in a certain level of malnutrition risk (low, moderate, and high) (see, e.g., Syuhada and Nur'aini [22] for finding some distributions related to severity and frequency risk). The ultimate problem is the statistical modeling of HaM data. It is common that finding and determining risk factors may be approached by the regression model, whilst the level of malnutrition risk is classified by logistic regression. By having these statistical analysis, we then determine in which level the HaM risk attached to the patients.

## 2. Methods

### 2.1. Study Design

This study employed a cross-sectional design using the medical records of patients from 1 month to 18 years of age who were hospitalized in a top referral hospital in Indonesia for at least 72 hours. Patients with tumors and organomegaly, fluid retention, and dehydration were excluded from the study.

The study was conducted in a top referral hospital located in the capital city of Indonesia (the hospital's name could not be mentioned due to the research contract between the researcher and the hospital). The patients who were admitted to this hospital originate from varied provinces in Indonesia. There were five wards related to children, namely, neonatology ward, pediatric surgical ward, pediatric intensive care unit, pediatric emergency unit, and general pediatric ward.

The researchers collected patients' ID number who were hospitalized from January to December 2017 from pediatric wards. Then, the medical record officer located the available medical records and provided them to the researcher. The researcher started to collect the data according to the questionnaire previously developed by the researcher which consisted of all research variables. Following the screening process for the inclusion and exclusion criteria, as well as the completeness of data provided, 308 medical records were analyzed.

### 2.2. Study Variables

The data obtained included patients' identity (initials), date of admission and discharge from the hospital, date of birth, medical diagnosis, nutritional therapy, and weight on admission. Hospital-acquired malnutrition was decided through comparing patients' admission weight to their weights when they were discharged.

### 2.3. Statistical Analysis

Statistical analysis was conducted using SPSS version 22. A univariate analysis was conducted on the independent variable in this study to identify the characteristics of pediatric patients. The variables of sex, age, and weight were collected as numerical data. The numerical data were presented as mean, standard deviation, and kurtosis.

Meanwhile, other characteristics, that are age category, nutritional route, the existence of pain, dyspnea, nutritional status at admission, and deterioration status in the first three days, were also evaluated. Deterioration status was measured using the Nursing Early Warning System (NEWS), an instrument developed and used by the hospital to identify patients' worsening condition during hospitalization. Such variables considered as categorical data were then analyzed using a proportion test to measure the frequency and percentages of each category.

For inferential statistics, testing of independence by chi-square statistic was conducted to test whether or not there was a relationship between weight difference and risk characteristics. As for the predictive model, we carried out linear regression modeling, whilst risk scoring or malnutrition-at-risk (MaR) calculation was computed based on logistic regression.

### 2.4. Ethics Considerations

This study was approved by the Ethics Committee of Faculty of Medicine, Universitas Indonesia.

## 3. Results

### 3.1. Descriptive Statistics

**Figure 1:**
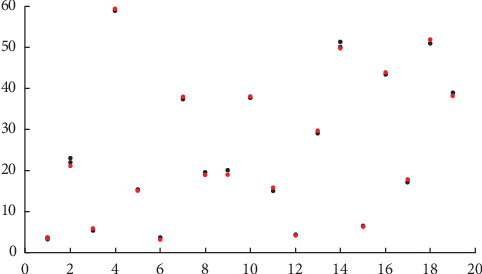
Daily weight on day 1 (red dot) and on other days (black dot) (*y*-axis) against the selected patient (*x*-axis); when the black dot appears, the weight on day *t* is different from that of day 1.

The case of HaM may be viewed statistically at first through a scatter plot.  Figure 1 shows the daily weight (*y*-axis) against the selected patients (*x*-axis) with red dot/mark denoting the patient's weight on day 1 (at admission). When there is a black dot/mark, it is an indication of weight difference between day *i*, *i* = 2,…, 5, and day 1; specifically, when the black dot position is below the red one, weight difference is negative (there is weight loss), i.e., the patients' weight decreases. For example, weight losses occur to patient numbers 3, 4, 7, 11, 13, 16, 17, and 18, whilst other patients have positive weight difference.

**Figure 2:**
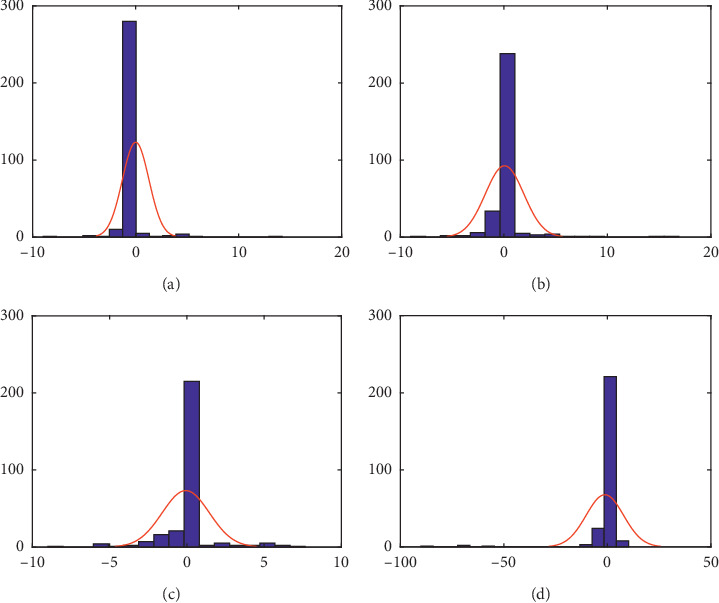
Histogram for weight difference (WeightDiff) between day *t*, for *t*=2,3,4, and the day of discharge (*D*), against day 1 with the normal curve. (a) WeightDiff 21, (b) WeightDiff 31, (c) WeightDiff 41, and (d) WeightDiff D1.

Figure 2 shows the statistical weight difference behavior with a reference of normal distribution through the histogram with a normal curve. Patients' weight difference is calculated for day *t*, for *t*=2,3,4, and the day of discharge (*D*), against day 1. Negative values of weight difference on day 3 and 4 against day 1 (WeightDiff 31 and WeightDiff 41) suggest potential HaM cases. Inappropriateness of normal distribution to the data also suggests the use of other heavy-tailed distributions.

Table 1 shows the summary of statistics (mean, standard deviation, and kurtosis) of patients' weight from day 1 to day 5 and the day of discharge. It also shows patients' weight on day 1 and day 2 for each level of age. Patients' weight on day 5 decreases by 0.21% compared with the weight on day 1 although the weight fluctuates during hospitalization. For each level of age, there is a small decrease in patients' weight, e.g., infant (0.22%), toddler (0.07%), and adolescence (0.02%). As for standard deviation, it is relatively high which shows the high spread of patients' weight. We observe, however, that the standard deviation is relatively small when patients' weight is classified as infant, toddler, preschool, school age, and adolescence. This spread is also described by its high kurtosis particularly for patients' weight in their categories (10.77 for infant, 37.56 for toddler, and 12.31 for preschool); note that this is greater than kurtosis for normal distribution which is 3.

We present the number of patients, in Table 2, for each level of risk factors or characteristics. Most pediatric patients we observed were male (56%), whereas patients were mostly school age. Few pediatric patients observed were diagnosed with cancer (46%). It is important to note that the number of patients is different during hospitalization: 308 patients on day 1 and day 2, 300 patients on day 3, 286 patients on day 4, and 260 patients on day 5. This is because some patients were discharged during the study.

It is interesting to how number of patients changes every day according to diet (in which regular food are mostly consumed by patients, about 60%–62%) and nutrition route (which is dominated by the oral nutrition route). Enteral and parenteral routes come in lower numbers, respectively. Most pediatric patients have no pain (more than 79%) during hospitalization and no dyspnea which is more than 81% from day 1 to day 5. According to the NEWS (Nursing Early Warning System) score, pediatric patients mostly have a green score (more than 80%) that indicates stable condition. As stated above, Moeeni et al. [15, 16], this condition, in fact, may have a high probability for the HaM case to occur.

### 3.2. Inferential Statistics

Table 3 presents the significant probability (*p* value) for testing the hypothesis of independence, i.e., whether there is an association between weight difference (category) on day *t* to day 1 and certain risk factors or characteristics. Our weight difference (WeightDiff) categories increased more than 2%, increased up to 2%, showed no weight difference, decreased up to 2%, and decreased more than 2%; note that the numbers “21” to “51” denote day 2 against day 1, etc. The statistic test is *χ*^2^. Risk factors of age, medical diagnosis, diet, nutrition route, pain, and dyspnea have a high possibility of significant factors for the case of HaM (since they have association) due to its probability of significance (*p* value) for less than 5 percent to the patients' weight.

We compute the probability of having weight decrease each day.  Table 4 provides the empirical probability of patients' weight increase or decrease (for each category) for all days during hospitalization. Such probability is computed simply by the ratio of the number of weight difference category and the total number of observations. The first two rows show positive weight increase (more than 2% and up to 2%, respectively), but the last two rows represent the decrease. There is a high probability for no weight difference during the patients' stay at the hospital, about 0.77 (mean during hospitalization).

### 3.3. Predictive Model: Linear Regression

Hospital-acquired malnutrition (HaM) risk, we have considered, is calculated via weight difference on day *t*, for *t*=2,3,4,5, relative to day 1. Risk factors or characteristics are obtained from both dependence testing of *χ*^2^ (chi-square) and testing mean. The results suggested us to propose the following regression model:

(1) $$\begin{array}{c} \text{weight difference}=\beta_{0}+\beta_{1}\cdot \text{sex}+\beta_{2}\cdot \text{age}+\beta_{3}\cdot \text{medical diagnosis}+\beta_{4}\cdot \text{diet}+\beta_{5}\cdot \text{nutrition route}+\\ +\beta_{6}\cdot \text{NEWS score}+\text{model innovation}, \end{array}$$

for *i*=1,2,…, *n*, where the assumption of normality for model innovation, or model error, has been used. The coefficients of such risk factors are displayed in Table 5. Testing the coefficients indicate that these risk factors affect the HaM case.

As stated before, the HaM case occurs when the patient weight, on day *t*, decreases more than 2%, relative to day 1 or admission. In doing so, we conducted a one-way analysis of variance (ANOVA) to test the weight difference on day *t* for each risk factor levels.  Table 6 shows the *p* value for such analysis of variance. For example, 0.069 is the *p* value of analysis of variance of testing the mean of weight difference on day 2 for all levels of age. The null hypothesis is that there is no difference in weight for all levels of age (infant, toddler, preschool, school age, and adolescence). By a given level of confidence of *α*=10%, we conclude that null hypothesis is rejected. In other words, there is weight difference for the risk factors or characteristics of age on day 2; (^*∗*^) and (^*∗∗*^) marks show that the corresponding null hypothesis is rejected at the level of 10% and 5%, respectively.

### 3.4. Risk Scoring: Logistic Regression

Table 7 shows the logistic regression score of risk classification: “0” for no HaM and “1” for HaM (dependent variable). However, independent variables such as sex, age, medical diagnosis, diet, nutrition route, and NEWS score are taken from the linear regression modeling result. We aim at finding a malnutrition alarm score for each risk factor level.

## 4. Discussion

HaM cases are observed and calculated during hospitalization regardless of the patients' nutritional status on day 1 (at admission). If a patient's weight tends to decrease at day *t*, for *t*=1,2,…, then a HaM case occurs. In other words, when a patient's weight difference has a negative value, “weight on day *t* is less than weight on day 1,” then there is an indication of an occurrence of the HaM case. To confirm an HaM case, according to Pacheco-Acosta et al. [13], the (negative) weight difference or loss must be greater than two percent. Later, we may call this as weight loss.

Patient weight difference distribution shows non-normal distribution. This may be indicated by its kurtosis which is greater than 3. There are possibilities to do data transformation as well by employing heavy-tailed distribution. Nonetheless, the histogram and normal curve of data may tell us the possibility of the HaM case due to negative values of weight difference shown on days 3 and 4. In addition, to compute the weight difference on each day, we employ a statistic of mean, and its spread is calculated via standard deviation and kurtosis. As stated before, high kurtosis indicates that patient's weight (or patient's weight difference) has a value that spreads quite far from its mean. In statistical theory, this features leptokurtic or heavy tailed.

The number of pediatric patients for each risk factor of characteristic is an important tool to detect a possible HaM case. For instance, in this study, the number of male patients is greater than the female patients. During hospitalization, it is likely that a HaM case or the first HaM case occurs among male patients [23]. If, in fact, HaM case(s) or the first HaM case is found to be a female patient, then it is possible that sex is potential to be a risk factor for the HaM case. The similar description also happens to the risk factor of age that has five factor levels.

For other risk factors, say diet, the number of patients with soft food or liquid food is lower in comparison to regular food, providing us with low possibility of an HaM case. This is because (more) severe illness or disease and patient condition have association with patient's diet. Thus, it is a potential risk factor for the HaM case to occur. In fact, our study shows low number of patients. Another risk factor such as nutrition route tells us on how low possibility of the HaM case for pediatric patients occurs with the oral nutrition route. This is supported by the study by Villares et al. [21].

To gain more accurately on what risk factors or characteristics affect the HaM case, we carry out an inferential statistics, namely, hypothesis testing. For each day during hospitalization, we compute weight difference, say WeightDiff 21, WeightDiff 31, WeightDiff 41, and WeightDiff 51. For risk factor levels, we aim to find out whether there is a mean difference of weight on a certain day for a certain risk factor. The risk factor of medical diagnosis, for example, has significant difference in mean for their levels: cardio, respiro, neuro, onco, infectious, and others. With the *p* value obtained, the *p* value is equal to 0.02, it is concluded that the weight difference on day 5 has a difference in mean significantly among the levels of medical diagnosis. The same conclusion is taken for the risk factors of age, diet, and nutrition route.

Having several risk factors as given above, it is important to compute their contribution to weight difference in a predictive model. A regression model is appropriate to calculate such contribution. We pay particular attention to the coefficient for the linear regression model. Such coefficients tell us the amount of certain risk factors' contribution to weight difference when the risk factor changes in one unit. For instance, the value of 0.03 for medical diagnosis and 0.48 for diet coefficients, respectively, inform us that when such risk factor changes one unit, the contribution of medical diagnosis to weight difference is 0.03 and 0.48. We do this computation for other risk factors: sex, age, diet, nutrition route, and NEWS score. Note that the risk factors of sex and NEWS score are included in this model since they are considered as important factors in the literature.

When weight difference is looked at in more detail, as stated before, we may find out how likely (in term of empirical probability) a HaM case will occur and in what day during hospitalization. We pay particular attention, of course, to weight loss that means negative weight difference of more than two percent. Pediatric patients, in general, tend to have the same weight with quite high probability: 0.8639 (day 2), 0.7825 (day 3), 0.7415 (day 4), and 0.6929 (day 5). For a particular day, say day 3 and day 4, patients' weight difference is below two percent with higher probability than the other amount of weight difference.

The result of the regression coefficient and the malnutrition alarm score for every level factor may be observed, and the total score is 134. From this total score, we need to find a limit or threshold value to determine whether or not a new patient has a higher risk to be malnourished. A 75% significant level was then chosen, and we have a threshold value of 100.5, see, e.g., Florkowski [24] and Mukuku et al. [25] for the ROC curve that covers sensitivity and specificity. This is called malnutrition-at-risk (MaR). If someone or a patient has a malnutrition total score of more than or equal to 100.5, we can classify that the person has a high risk to be malnourished. However, if someone/patient has a malnutrition total score of less than 100.5, we can state that the person has a low risk to be malnourished. The form of the malnutrition alarm score for HaM, including some factors/characteristics related, is given in the Appendix.

## 5. Conclusion

Hospital-acquired malnutrition cases related to some factors such as sex, age, medical diagnosis, diet, nutrition route, and NEWS score are analyzed. Accordingly, these factors were included in the calculation of risk scoring, called malnutrition-at-risk (MaR), to predict the potential risk of HaM. This score can be used to provide signals to health team members as to which patients need more attention on their nutritional status, or which patients have the possibility to experience nutritional status decline.

Some risk factors of HaM may be observed through (i) an aggregate model, e.g., Syuhada and Nur'aini [22], and/or (ii) stochastic modeling such as heteroscedastic processes, see, e.g., Syuhada [26]. For the latter modeling, we may look at data as time increases and do bootstrap analysis since the number of data is very low. The problem of forecasting the risk score for future malnutrition incident may be carried out by numerical analysis of coverage probability conditional on previous weight difference or return.

When a patient has been assigned for a certain MaR score, we may be able to calculate the probability of the changing state, from high (low) risk to low (high) risk or from low (high) risk to high (low) risk via the Markov chain. This is called transition probability.

An important aspect of collecting data for HaM is that it is not well recorded. This may be due to human error in data recording. Hospital staff are encouraged to be aware for this matter.

## Figures and Tables

**Table 1:** Summary of statistics for patients' (i) weight (from day 1 to day 5 and the day of discharge) and (ii) weight on day 1 and day 2 for each level of age.

		Mean	SD	Kurtosis
Weight	Day 1	21.063	15.158	3.56
	Day 2	21.068	15.154	3.55
	Day 3	21.138	15.127	3.55
	Day 4	21.100	15.226	3.56
	Day 5	21.018	15.568	3.57
	Day *D*	21.116	15.168	3.54

Weight on day 1	Infant	5.349	2.158	10.77
	Toddler	10.166	4.352	37.56
	Preschool	14.390	3.452	12.31
	School age	26.384	8.943	3.34
	Adolescence	43.270	12.440	2.24

Weight on day 2	Infant	5.337	2.162	10.75
	Toddler	10.159	4.357	37.45
	Preschool	14.395	3.443	12.44
	School age	26.413	8.927	3.36
	Adolescence	43.260	12.410	2.25

**Table 2:** Number of patients due to risk factors or characteristics related to HaM.

Patient characteristics	Day 1	Day 2	Day 3	Day 4	Day 5
*Sex*					
Male	173				
Female	135				

*Age*					
Infant	46				
Toddler	58				
Preschool	62				
School age	82				
Adolescence	60				

*Medical diagnosis*					
Cardiac	6				
Respiratory	46				
Neurology	16				
Oncology	143				
Infectious	8				
Others	89				

*Diet*					
Regular food	187	187	186	178	159
Soft food	21	21	18	17	13
Liquid food	83	88	90	85	83
Other foods	17	12	6	6	5

*Nutrition route*					
Oral	231	234	228	217	195
Parenteral	34	32	31	28	24
Enteral	43	42	41	41	41

*Pain*					
Yes	65	59	60	46	47
No	243	249	240	240	213

*Dyspnea*					
Yes	59	56	52	46	38
No	249	252	248	240	221

*NEWS score*					
Green	258	260	260	253	231
Yellow	47	46	39	31	26
Orange	3	2	1	2	3
Red	0	0	0	0	0

Number of patients	308	308	300	286	260

**Table 3:** Significant probability (*p* value) of patients' weight difference (WeightDiff) according to risk factors or characteristics.

	WeightDiff 21	WeightDiff 31	WeightDiff 41	WeightDiff 51
Sex	0.432	0.462	0.226	0.636
Age	0.018^*∗*^	0.009^*∗*^	0.11	0.32
Medical diagnosis	0^*∗*^	0.007^*∗*^	0.14	0.02^*∗*^
Diet	0^*∗*^	0^*∗*^	0.037^*∗*^	0.302
Nutrition route	0.001^*∗*^	0.005^*∗*^	0.083^*∗∗*^	0.114
Pain	0.065^*∗∗*^	0.394	0.113	0.09^*∗*^
Dyspnea	0.628	0.21	0.495	0.038^*∗*^
NEWS score	0.536	0.337	0.26	0.137

^*∗*^ *p* value is less than 5 percent.

**Table 4:** Empirical probability of weight increase or decrease for pediatric patients.

WeightDiff category	Day 2	Day 3	Day 4	Day 5
Increase more than 2%	0.028	0.042	0.051	0.056
Increase up to 2%	0.015	0.023	0.030	0.044
No weight difference	0.864	0.783	0.742	0.693
Decrease up to 2%	0.076	0.120	0.126	0.131
Decrease more than 2%	0.020	0.033	0.052	0.089

**Table 5:** Regression coefficients of HaM risk factors.

Risk factors	Sex	Age	Diagnosis	Diet	Nutrition route	NEWS score
Coefficients	−0.21	0.07	0.03	0.48	−1.41	−0.04

**Table 6:** *p* value for one-way ANOVA for weight difference on day *t* for all risk factor levels.

	WeightDiff 21	WeightDiff 31	WeightDiff 41	WeightDiff 51
Sex	0.641	0.279	0.459	0.624
Age	0.069^*∗*^	0.977	0.82	0.419
Medical diagnosis	0.892	0.512	0.567	0^*∗*^
Diet	0.130	0.003^*∗∗*^	0^*∗∗*^	0.502
Nutrition route	0.328	0.014^*∗∗*^	0.026^*∗∗*^	0.230
Pain	0.940	0.966	0.37	0.103
Dyspnea	0.162	0.289	0.484	0.859
NEWS score	0.308	0.101	0.068^*∗*^	0.753

**Table 7:** Malnutrition alarm score based on the logistic regression coefficient for risk factors or characteristics.

Characteristics	Score
*Sex*	
Girl	0
Boy	2

*Age*	
Infant	21
Toddler	18
Preschool	5
School age	2
Adolescence	0

*Medical diagnosis*	
Cardio	0
Respiro	16
Neuro	3
Onco	3
Infectious	19
Others	16

*Diet*	
Regular	0
Soft	4
Liquid	13
Others	1

*Nutrition route*	
Oral	1
Parenteral	3
Enteral	2

*NEWS score*	
Green	3
Yellow	2
Orange	0
Red	0

## Data Availability

The pediatric patient data used to support the findings of this study have not been made available due to unavailable permission from the hospital where the study was conducted.

## Appendix

### Malnutrition Alarm

  Patients name: ____________
  Admission date: ____________
  Weight on Admission: __________
  Weight on day 2: ___________

  MaR > 45.5: High risk
  MaR ≤ 45.5: Low risk
